# PARP9 drives the malignant progression of pancreatic cancer cells by regulating apoptosis, DNA damage, and multidrug efflux systems

**DOI:** 10.3389/fcell.2025.1694345

**Published:** 2025-11-21

**Authors:** Xing Wang, Xiaolong Liu, Yulan Li

**Affiliations:** 1 The First Clinical Medical College of Lanzhou University, Lanzhou, China; 2 Department of Anesthesiology, The First Hospital of Lanzhou University, Lanzhou, China

**Keywords:** pancreatic cancer, Parp9, LOXL2, targeted therapy, multidrug efflux systems

## Abstract

**Background:**

Targeted therapy is a potent strategy for the treatment of advanced and metastatic cancers, with pancreatic cancer (PC) being one of the leading causes of cancer-related deaths worldwide. In order to resolve the limitations of existing targeted agents, there is an urgent need to find new targets and therapeutic strategies. Poly (ADP-ribose) polymerase 9 (PARP9) is aberrantly expressed in a variety of tumors. However, its relationship with PC has not been fully investigated. Lysyl oxidase like 2 (LOXL2) is potential therapeutic targets in challenging PC, which contributes to the malignant progression of PC and poor prognosis.

**Methods:**

Cell lines with PARP9 knockdown or overexpression were established by lentiviral transfection, while LOXL2 was overexpressed by plasmid, and we validated the effect of PARP9 on apoptosis and DNA damage in PC cells using flow cytometry, comet assay, and western blots. The changes in downstream targets and signaling pathways after PARP9 expression changes were analyzed by RNA sequencing and protein blotting analysis. Finally, the relationship between PARP9 and LOXL2 was analyzed by immunoprecipitation, and the multidrug efflux system was determined by boinformatics analysis and western blots.

**Results:**

PARP9 and LOXL2 were highly expressed in PC tissues and were associated with poor prognosis. PARP9 knockdown significantly inhibited the proliferation, invasion, and migration of PC cells, while also promoting apoptosis, increasing DNA damage, and inhibiting multidrug efflux systems. Meanwhile overexpression of LOXL2 reduced apoptosis and DNA damage, and drug sensitivity in PC cells caused by PARP9 knockdown. The therapeutic process of PARP9 in PC may be achieved through the synergistic action of LOXL2 and PI3K/AKT signaling pathway.

**Conclusion:**

Our study reveals a potential link between PARP9 and PC, and targeting PARP9 and LOXL2 in monotherapy or combination therapy may provide novel strategies to advanced PC.

## 1 Introduction

Pancreatic cancer (PC) is a highly malignant tumor of the digestive system with an extremely poor prognosis, often referred to as the “king of cancers”. By 2022, the global incidence and mortality rates continued to rise, with an estimated 511,000 new cases and 467,000 deaths, representing 4.8% of cancer-related mortality, ranking sixth worldwide (Bray et al., 2024). Projections indicate that by 2050, the incidence rate is expected to rise to 18.6 per 100,000 population with an annual growth rate of 1.1%, signifying a substantial impending public health burden (Hu et al., 2021). Current evidence indicates that first-line gemcitabine-based chemotherapy improves prognosis and elevates the 5-year overall survival rate from 3% to 8% in advanced PC (S. Zeng et al., 2019). Nevertheless, the extensive application of this regimen has led to the rapid emergence of chemoresistance, which now constitutes a major therapeutic obstacle in PC management (Amrutkar & Gladhaug, 2017). Gemcitabine resistance arises from multiple factors, including activation of the multidrug efflux system (Gu et al., 2022; Y. Wu et al., 2025), enhanced DNA repair pathways (Ding et al., 2019; Sheikh et al., 2010), and dysregulation of apoptosis (Shi et al., 2002).

Recent advancements in targeted therapy have also shown promise in PC treatment. These therapies inhibit tumor growth and dissemination by interfering with tumor-specific molecular pathways, offering superior selectivity and therapeutic potential compared to conventional cytotoxic chemotherapy. Poly (ADP-ribose) polymerase (PARP), a nuclear enzyme family comprising 18 members, catalyzes the transfer of ADP-ribose moieties to target proteins (Amé et al., 2004). As a crucial DNA repair enzyme, PARP maintains genomic integrity and regulates apoptosis (Bourlon et al., 2024). Clinically approved PARP1-targeting agents include Olaparib, Veliparib, and Niraparib. Current research focuses on enhancing PARP inhibitor efficacy through novel compound development and combination therapies. A phase II trial comparing Olaparib-pembrolizumab combination versus Olaparib monotherapy in metastatic PC maintenance treatment demonstrated extended median progression-free survival from 7 months to 11.7 months (Chung et al., 2021). However, acquired resistance to PARP inhibitors has emerged as a significant clinical challenge (Lord and Ashworth, 2017). The multidrug efflux is one of the causes of PARPi resistance. The ATP binding cassette subfamily B member 1 (ABCB1) gene, also known as the multidrug resistance gene, encodes the p-glycoprotein efflux pump, which ultimately reduces the amount of PARPi drugs available to the cell, leading to reduced efficacy and PARPi resistance, especially in BRCA1 gene-deficient breast and ovarian cancer cell lines (Rottenberg et al., 2008; Vaidyanathan et al., 2016). At the same time, ABCB1 inhibitors (e.g., verapamil and elacridar) can reverse ABCB1-mediated resistance in ovarian cancer cells treated with olaparib (Vaidyanathan et al., 2016).

PARP9 demonstrates upregulated expression across multiple malignancies and exhibits functional relevance to tumorigenesis, progression, and therapeutic response. In PC subsets, IFNβ-induced upregulation of NAD + -consuming enzymes PARP9, PARP10, and PARP14 enhances tumor sensitivity to nicotinamide phosphoribosyltransferase inhibitors (Moore et al., 2021). Nevertheless, the precise relationship between PARP9 and PC remains incompletely characterized. Systematic integration of existing knowledge about PARP9’s roles in other tumor types and functional elucidation in PC may reveal novel therapeutic targets for this disease.

In this study, we initially identified PARP9 overexpression in PC specimens, with elevated expression correlating with adverse clinical outcomes in PC patients. Furthermore, PARP9 overexpression potentiated malignant biological behaviors of PC cells both *in vitro* and *in vivo*. Mechanistically, PARP9 interacts with LOXL2 to confer resistance against apoptosis, DNA damage, and drug sensitivity in PC cells through activation of the PI3K/AKT signaling pathway. Collectively, this study delineates the regulatory mechanism of PARP9-mediated LOXL2 modulation in PC and proposes a novel therapeutic target for intervention.

## 2 Materials and methods

### 2.1 Bioinformatics analysis

GEPIA (Gene Expression Profiling Interactive Analysis) was used to examine the expression of PARP9 in TCGA + GTEx and overall survival (OS) or disease-free survival (DFS) and the correlation between two genes in PC patients. The Gene Expression Omnibus (GEO) dataset GSE15471 was utilized to analyze PARP9 expression in paired tissue samples and GSE140077 was employed to analyze the alterations associated with gemcitabine chemoresistance in PC cells. Protein expression analysis was performed using the Ualcan database (https://ualcan.path.uab.edu/analysis-prot.html). The Kaplan-Meier plotter (http://kmplot.com/analysis/) was employed to evaluate the association between PARP9 or LOXL2 expression and OS or DFS in PC patients.

### 2.2 Human tissue samples

Human PC tissue samples and matched adjacent non-tumor tissues were collected from three patients diagnosed with PC based on histopathological evaluation, who underwent surgical resection at the First Hospital of Lanzhou University from 2024/11/1 to 2025/1/22 (LDYYLL 2024-686). Matched adjacent non-tumor tissues were obtained from the region farthest from the tumor margin (>5 cm) in each resected specimen. All samples were immediately frozen in liquid nitrogen after resection and stored at −80 °C. None of the patients had received prior local or systemic therapies before surgery. The study was approved by the Ethics Committee of Lanzhou University.

### 2.3 Immunohistochemistry (IHC)

Tissues were fixed, dehydrated, and embedded in paraffin. 4 μm thick sections were deparaffinized with xylene, rehydrated through a graded ethanol series, and subjected to antigen retrieval using EDTA buffer. Endogenous peroxidase activity was blocked with 3% H_2_O_2_, followed by incubation with 5% BSA (Servicebio, China) for nonspecific site blocking. Primary antibodies were applied and incubated overnight at 4 °C. After washing with PBST, sections were incubated with species-matched secondary antibodies at room temperature for 2 h. Color development was performed using a diaminobenzidine working solution. Sections were subsequently dehydrated through a graded ethanol series, cleared in xylene, and mounted with neutral resin.

### 2.4 Cell culture

The normal human pancreatic ductal epithelial cell line hTERT-HPNE and PC cell lines (PANC-1, AsPC-1, CFPAC-1, PA-TU-8988T) were obtained from the Cell Bank of the Chinese Academy of Sciences. hTERT-HPNE, PANC-1, and PA-TU-988T cells were cultured in DMEM medium (Servicebio, China) supplemented with 10% fetal bovine serum (Biochannel, China) and 1% penicillin. CFPAC-1 cells were maintained in IMDM medium (Servicebio, China) containing 10% FBS and 1% penicillin. AsPC-1 cells were grown in RPMI-1640 medium (Servicebio, China) with 10% FBS and 1% penicillin. All cell lines were incubated at 37 °C in a humidified atmosphere of 5% CO_2_ and 95% air using a cell culture incubator (Thermo, USA).

### 2.5 RNA extraction and qRT-PCR experiments

Total RNA was extracted from PC cells using TRIzol reagent (Servicebio, China). RNA concentration and purity were measured with a NanoDrop spectrophotometer (Servicebio, China), and samples with A260/A280 ratios between 1.8 and 2.0 were retained for downstream analysis. cDNA synthesis was performed using the PrimeScript™ RT Reagent Kit with gDNA Eraser (Takara, Japan), followed by quantitative real-time PCR (qPCR) amplification with Premix Ex Taq™ II (Takara, Japan) on a Bio-Rad CFX96 (USA) real-time PCR system. mRNA expression levels were normalized to GAPDH mRNA expression. The sequences of the primer pairs are shown in Supplementary Table S1.

### 2.6 Stabilized cell line and plasmid construction and transfection

Lentiviral vectors, including the negative control lentivirus (vector and PARP9 NC), PARP9 overexpressing lentivirus (PARP9 OE), and PARP9 knockdown lentiviruses (PARP9 SH), were purchased from GENERAL BIOL (China). The plasmid pCMV-LOXL2 (human)-3×FLAG-Neo for overexpression of human LOXL2 was designed and synthesized by MiaoLingBio (Wuhan, China; Catalog #: P49431). Stable transfectants were selected using puromycin dihydrochloride (Biosharp, China).

### 2.7 Western blot

The sample tissues and cultured cells were lysed with RIPA buffer (Beyotime, China) supplemented with PMSF (Servicebio, China) and then centrifuged at 12,000 g for 15 min at 4 °C. Protein concentrations were quantified using a BCA Protein Assay Kit (Servicebio, China). Proteins were denatured in SDS-PAGE loading buffer (Beyotime, China) at 100 °C for 10 min and subsequently separated on 7.5%–15% SDS-polyacrylamide gels. β-actin and GAPDH were used to normalize the level of protein expression. Densitometric analysis was performed with ImageJ software. The raw data from Western blot in this study were uploaded to Supplementary Material 1.

### 2.8 CCK-8 proliferation assay

PC cells were seeded into 96-well plates at 3,000 cells/well. At 0, 24, 48, and 72 h, CCK-8 reagent (APExBIO Technology LLC, China) was added and incubated for 2 h at 37 °C. Absorbance was measured at 450 nm using a microplate reader (Thermo, USA).

### 2.9 Wound healing

PC cells were seeded into 6-well plates and cultured until reaching over 90% confluency. After 48 h, the wound area was measured using an inverted microscope (Olympus, Japan) and the cell migration rate was calculated.

### 2.10 Colony formation assay

Cells were seeded into 6-well plates at a density of 1 × 10^3^ cells/well and cultured until colony formation. Colonies were washed, fixed with methanol for 30 min, and stained with crystal violet for 30 min at room temperature.

### 2.11 Transwell experiment

Cell migration was assessed using transwell chambers (Corning, USA). Briefly, 5 × 10^4^ cells suspended in serum-free medium were plated in the upper chamber, and at the same 800 μL of complete medium was added to the lower chamber. After 24 h of incubation, cells in the upper chamber were gently removed with a cotton swab. Migrated cells on the lower membrane were fixed with methanol, stained with crystal violet, and imaged.

### 2.12 Flow cytometry for apoptosis

Cells were harvested, resuspended in binding buffer, and stained with 5 μL Annexin V-FITC and 10 μL propidium iodide (PI) (Beyotime, China). After 30-min incubation in the dark at room temperature, samples were analyzed using a flow cytometer (Beckman, USA).

### 2.13 Comet assay

Single-cell suspensions were embedded in agarose gel on slides. Following cell lysis, slides were placed in a horizontal electrophoresis chamber containing alkaline electrophoresis buffer to unwind DNA. Electrophoresis was performed under specific voltage/current conditions to allow damaged DNA fragments to migrate toward the anode. Slides were neutralized, stained, and imaged**.**

### 2.14 RNA sequencing and data analysis

We employed the CFPAC-1 cell line for RNA sequencing. Total RNA was extracted from PARP9 knockdown or control CFPAC-1 cells using TRIzol reagent (Servicebio, China). RNA quality was verified before library preparation. Sequencing was performed on the Illumina NovaSeq 6,000 platform by Tsingke Technology (China), with subsequent data analysis conducted by their bioinformatics team.

### 2.15 Immunofluorescence staining

PARP9 was knocked down in CFPAC-1 cells using a lentivirus to induce DNA damage. Following digestion, cells were seeded at a density of ∼1 × 10^4^ cells per well onto sterile circular coverslips in 24-well plates. Cells were cultured in 500 μL of complete medium at 37 °C with 5% CO_2_ overnight to ensure adhesion. Subsequently, cells were fixed with 4% PFA for 15 min, permeabilized with 0.2% Triton X-100 for 10 min, and blocked with 1% BSA. Then, cells were incubated overnight at 4 °C with a mixture of primary antibodies: LOXL2 and γH2AX. After washing, samples were incubated for 2 h at room temperature in the dark with the fluorescently labeled secondary antibody (Alexa Fluor 488 and Cy3) diluted 1:100. Nuclei were counterstained with DAPI. Images were acquired using a fluorescence microscope (Olympus, Japan).

### 2.16 Subcutaneous xenograft model

Eighteen 5–6-week-old female BALB/c nude mice (Beijing Vital River Laboratory Animal Technology Co., Ltd, China) were randomly divided into six groups (Vector (PANC-1), PARP9 OE (PANC-1), PARP9 NC (CFPAC-1, Aspc-1), NC KD (CFPAC-1, Aspc-1) groups). Mice were subcutaneously inoculated with 1 × 10^6^ stably transfected cells in the axillary region. Tumor dimensions were measured weekly for 4 weeks. Animals were euthanized via isoflurane overdose, and tumors were harvested for immunohistochemical (IHC) analysis. All procedures were approved by the Ethics Committee of the First Hospital of Lanzhou University (LDYYLL 2024-686).

### 2.17 Co-immunoprecipitation (Co-IP)

Cells were cultured in 10-cm dishes (minimum cell density: 1 × 10^7^ cells/dish) and harvested at full confluence. CFPAC-1 cells were lysed using ice-cold lysis buffer supplemented with protease inhibitors, followed by 10-min on-ice incubation. Lysates were centrifuged at 14,000 rpm for 15 min at 4 °C to collect supernatants. Protein samples were incubated with target-specific antibodies overnight at 4 °C. Protein A/G beads (ACE, China) pre-equilibrated with lysis buffer were subsequently added to form immunocomplexes via 1-h incubation at room temperature. Bead-bound complexes were washed extensively and subjected to Western blot analysis.

### 2.18 Statistical analysis

All statistical analyses were performed using SPSS v22.0 (IBM, USA) and GraphPad Prism 8.0.2 (GraphPad Software, USA). Continuous variables were tested for normality using the Shapiro-Wilk test and for homogeneity of variances using Levene’s test. Normally distributed data are presented as mean ± SD. For comparisons between two groups, a two-tailed Student’s t-test was used for parametric data, and the Mann-Whitney U test for non-parametric data. Survival analysis was performed using the Kaplan-Meier method, and differences between curves were assessed with the log-rank test. Statistical significance was set at a two-sided P value <0.05.

## 3 Result

### 3.1 PARP9 modulates apoptosis and DNA damage response in PC cells

**FIGURE 1:**
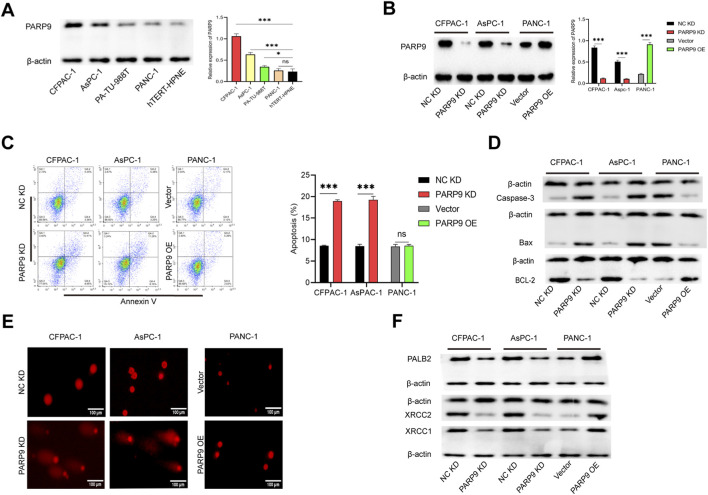
PARP9 modulates apoptosis and DNA damage response in PC cells. **(A)** The expression of PARP9 in four PC cell lines and normal pancreatic ductal epithelial cells four PC cell lines was examined by Western blot. **(B)** PARP9 was knocked down in CFPAC-1and AsPC-1 cells and overexpressed in cells, and the knockdown and overexpression efficiency were verified by Western blot. **(C)** Effect of PARP9 on the rate of apoptosis in PC cells. **(D)** Western blot analysis was performed to assess the expression levels of Caspase-3, Bax, and Bcl-2 after alteration of PARP9. **(E)** DNA damage in PC cells significantly increased post-PARP9 knockdown as determined by comet assay. **(F)** The anti-DNA damage capacity of PC cells decreased markedly following reduction of PARP9 expression. ***p < 0.001.

We found that PARP9 expression was elevated in PC and was associated with poor PC prognosis (Supplementary Figure S1)**.** To investigate the functional role of PARP9 in pancreatic carcinogenesis, we selected four human PC cell lines with distinct endogenous PARP9 expression profiles: CFPAC-1, AsPC-1, and PA-TU-8988t cells, which significantly exhibited relatively high basal PARP9 expression and PANC-1 cells, which did display relatively low PARP9 expression levels (Figure 1A). Initial screening for the lentivirus with the highest knockdown efficiency among the three knockdown lentivirus was performed using PCR (Supplementary Figure S2). And then, stable PARP9-transfected cell lines were established using lentiviral vectors of the highest knockdown efficiency, and transfection efficiency was rigorously validated by Western blot analysis under standardized experimental conditions (Figure 1B). PARP9 overexpression enhanced proliferation, invasive and migratory capacity, but diminished after knockdown (Supplementary Figure S3). Our study explored the functional relationship between PARP9 and the regulation of PC cells apoptosis. It is worth noting that knocking down PARP9 increased the apoptosis rates of CFPAC-1 and AsPC-1, while its overexpression in PANC-1 did not show significant changes (Figure 1C). Western blot analysis confirmed these observations. PARP9 knockdown was associated with upregulated expression of pro-apoptotic markers Caspase-3 and Bax, concurrent with downregulation of the anti-apoptotic protein Bcl-2. Conversely, overexpression of PARP9 induced diametrically opposed expression patterns in these apoptotic regulators (Figure 1D; Supplementary Figure S4A). DNA damage assessment via comet assay revealed pronounced tail formation under PARP9 knockdown conditions, indicative of genomic instability exacerbation. However, PARP9 overexpression did not significantly modulate this DNA damage phenotype (Figure 1E). Western blot profiling demonstrated that PARP9 depletion substantially attenuated expression of three critical DNA damage response genes - PALB2, XRCC2, and XRCC1, but these proteins showed enhanced expression after overexpression of PARP9 (Figure 1F; Supplementary Figure S4B). This indicated that the ability of PC cells to resist DNA damage decreased with decreasing levels of PARP9, but increased with its overexpression. We also measured the expression level of phosphorylated H2A histone family member X (γH2AX), a key indicator reflecting chromatin-level changes during DNA double-strand break and repair. The results demonstrated that PARP9 knockdown significantly increased γH2AX levels (Supplementary Figure S5A, B).

### 3.2 PARP9 drives PC cells progression via PI3K/AKT signaling

**FIGURE 2:**
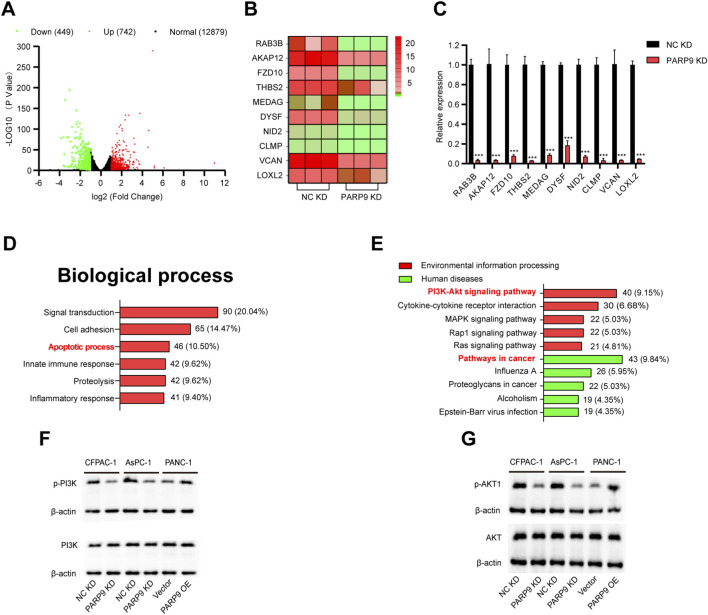
PARP9 drives PC cells progression via PI3K/AKT Signaling. **(A)** Volcano plot of differentially expressed mRNA. **(B)** Heat maps depicting the top ten genes with the highest reduction ratios among the differential genes. **(C)** Quantitative reverse transcription PCR (qRT-PCR) validated the ten genes exhibiting the greatest reduction ratios. **(D)** Effect of PARP9 on the rate of apoptosis in PC cells. **(E)** Enrichment pathways of differentially expressed genes were analyzed using KEGG analysis. Western blot analysis was performed to examine changes in PI3K **(F)** and AKT **(G)** expression following alterations in PARP9 levels. ***p < 0.001.

To further investigate the role of PARP9 in PC, volcano plots, which were generated based on, identified 1,191 differentially expressed genes (742 upregulated, 449 downregulated; fold change ≥2, P < 0.05) (Figure 2A). A heat map was constructed by analyzing the top ten genes with the most significant percentage of downregulation (Figure 2B). qRT-PCR validated that these 10 genes were also downregulated (Figure 2C). Analysis of the effects of PARP9 on biological process indicated that alterations were predominantly focused on the signal transduction, cell adhesion, and apoptotic process (Figure 2D). Further analysis indicated that in human diseases, differentially genes were mainly associated with cancer and that in terms of Environmental information, differential gene enrichment mainly focused on the PI3K/AKT signaling pathway and cytokine-cytokine receptor interactions (Figure 2E). Western blot confirmed PARP9 depletion reduced phosphorylated PI3K/AKT without affecting total protein levels, while PARP9 overexpression significantly enhanced the expression of p-PI3K and p-AKT without altering their total protein quantities (Figures 2F,G).

### 3.3 PARP9 knockdown inhibits tumor growth *in vivo*

**FIGURE 3:**
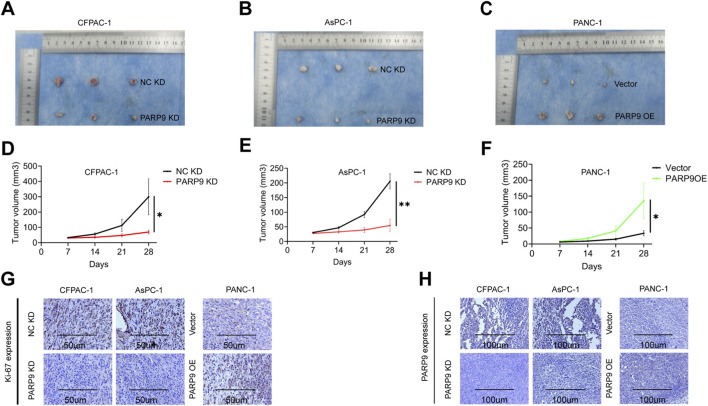
PARP9 knockdown inhibits tumor growth *In Vivo*. Nude mice were subcutaneously injected with NC KD or PARP9 KD CFPAC-1 **(A)** and AsPC-1 **(B)** cells. **(C)** Nude mice were subcutaneously injected with PANC-1 cells transfected with Vector or PANC-1 cells overexpressing PARP9. Tumor sizes were measured after sizeable tumor formation (day 7) following injection with NC KD or PARP9 KD CFPAC-1 **(D)** and AsPC-1 **(E)** cells. **(F)** Tumor sizes were measured after sizeable tumor formation (day 7) following injection with PANC-1 cells transfected with Vector or PANC-1 cells overexpressing PARP9. Inoculated mice were sacrificed on days 28 (PARP9 OE and Vector groups, or NC KD and PARP9 KD groups) and the tumors were excised for analysis (n = 3). Representative immunohistochemical staining of Ki-67 **(G)** and PARP9 **(H)** in xenograft tumors from nude mice in the NC KD and PARP9 KD, and Vector and PARP OE groups. Scale bar, 100 μm. NC, negative control; PARP, Poly (ADP-ribose) polymerase; KD, knockdown; SH, short hairpin; OE, overexpressing. *p *<* 0.05, **p < 0.01.

To investigate the in vivo functional consequences of PARP9 in PC progression, we conducted a subcutaneous xenograft tumorigenesis assay by implanting PC cell lines into immunocompromised nude mice. Stable cell lines were generated through lentiviral transduction, including PARP9 knockdown and negative control variants of CFPAC-1 and AsPC-1 cells, along with PARP9 overexpressing and empty vector-transfected PANC-1 cells. Comparative analysis of tumor growth status revealed substantial suppression of xenograft development in PARP9 knockdown groups relative to negative controls across both CFPAC-1 and AsPC-1 models and conversely, PARP9 overexpressing PANC-1 xenografts exhibited enhanced tumorigenic potential compared to vector-control counterparts (Figures 3A–F), and knockdown or overexpression of PARP9 had no effect on body weight in mice (Supplementary Figure S6). The IHC results of Ki-67 in the PARP9 KD group were significantly reduced, while those in the PARP9 OE group were significantly increased, confirming that PARP9 knockout can significantly inhibit the progression of xenograft tumors (Figures 3G,H).

### 3.4 PARP9 interacts with LOXL2 to promote tumorigenesis

**FIGURE 4:**
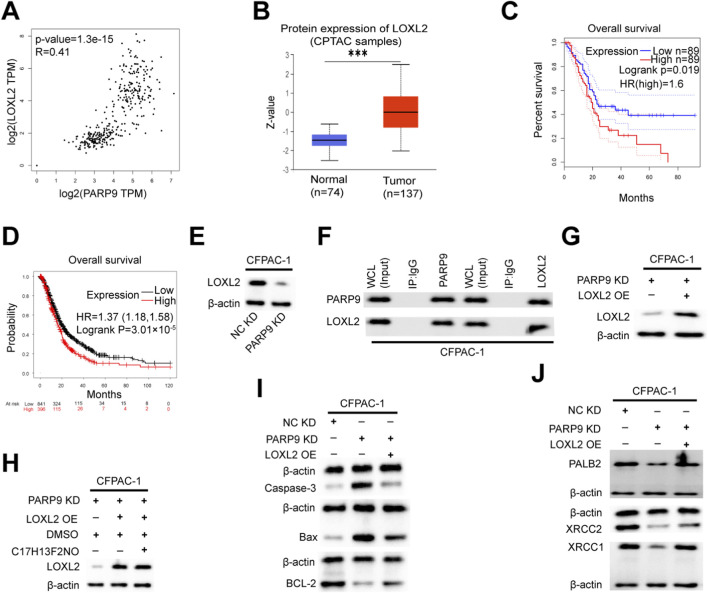
PARP9 interacts with LOXL2 to promote tumorigenesis. **(A)** The correlation between PARP9 and LOXL2 was validated using an online database. **(B)** The ualcan database was utilized to assess the expression of LOXL2 protein in unpaired samples from CPTAC. **(C,D)** Kaplan-Meier analysis indicated that high LOXL2 expression correlated with poorer overall survival compared to low LOXL2 levels in PC patients. **(E)** At the protein level, it was confirmed that LOXL2 levels decreased concomitantly with diminishing PARP9 expression in CFPAC-1 cells. **(F)** Co-immunoprecipitation (COIP) confirmed the interaction between PARP9 and LOXL2 in CFPAC-1 cells. **(G)** The overexpression of LOXL2 was verified through Western blot analysis in CFPAC-1 cells. **(H)** At the protein level, it was confirmed that the level of LOXL2 increases with the increase of PARP9 expression, and the PI3K/AKT signaling pathway inhibitor (C17H13F2NO) does not affect the expression of LOXL2 in CFPAC-1 cells, after treating CFPAC-1 cells with 2 μM inhibitor for 48 h. **(I)** Changes in apoptotic proteins in CFPAC-1 cells were assessed via a response experiment. **(J)** The response experiment also confirmed alterations in anti-DNA damage proteins within CFPAC-1 cells. NC, negative control; PARP, Poly (ADP-ribose) polymerase; KD, knockdown; SH, short hairpin; OE, overexpressing; Whole Cell Lysates, WCL. ***p < 0.001.

To further investigate the pathogenic mechanism of PARP9, we examined the correlation between PARP9 and the top ten genes with the most significant percentage of downregulation using the GEPIA database. This analysis revealed a significant positive correlation between PARP9 and LOXL2 expression (*R =* 0.41, P < 0.01) (Figure 4A). Comparative evaluation of clinical specimens demonstrated markedly elevated LOXL2 protein levels in PC tissues compared to normal pancreatic tissues (Figure 4B). Survival analysis further indicated that patients with high LOXL2 expression exhibited significantly poorer OS (Figures 4C,D). Previous study had shown that LOXL2 promoted PC growth of PC *in vivo* and *in vitro* as demonstrated by a subcutaneous xenograft, CCK-8 proliferation assay, colony formation assay and transwell experiment (Li et al., 2021). At the molecular level, Western blot analysis demonstrated the relationship between PARP9 and LOXL2 expression. PARP9 knockdown in PC cells resulted in reduced LOXL2 protein levels (Figure 4E). To explore the physical interaction between PARP9 and LOXL2, endogenous immunoprecipitation assays confirmed their direct binding in PC cells (Figure 4F). Functional rescue experiments were performed by transfecting PARP9 knockdown CFPAC-1 cells with a LOXL2-expressing plasmid, with successful overexpression confirmed by Western blotting (Figure 4G). Notably, pharmacological inhibition of the PI3K/AKT signaling pathway using the C17H13F2NO (PI3K/Akt/mTOR-IN-2, KKL MED, USA) did not alter LOXL2 expression, suggesting that PARP9 regulates LOXL2 independently of this pathway (Figure 4H). Remarkably, LOXL2 overexpression in PARP9 knockdown cells significantly attenuated apoptosis (Figure 4I) and enhanced resistance to DNA damage (Figure 4J), partially reversing the tumor-suppressive effects of PARP9 knockdown. At the same time, bioinformatic analysis revealed that PARP9 expression was positively correlated with the expression of PALB2 (R = 0.68, P < 0.01), XRCC2 (R = 0.47, P < 0.01), and XRCC1 (R = 0.71, P < 0.01). Similarly, LOXL2 expression was positively correlated with the expression of PALB2 (R = 0.53, P < 0.01), XRCC2 (R = 0.45, P < 0.01), and XRCC1 (R = 0.47, P < 0.01) (Supplementary Figure S7). In addition, the expression levels of homologous recombination genes (BRCA1, BRCA2, RAD51, RAD54) and non-homologous end joining genes (KU70, KU80, DNA-PKcs, XRCC4, LIG4, and XLF) were significantly positively correlated with the expression of PARP9 and LOXL2 (All P < 0.001, Supplementary Figures S8-S9). Moreover, research indicated that suppression of the PI3K/AKT pathway impaired the DNA repair mechanisms mediated by homologous recombination (Iida et al., 2024; Zhou et al., 2023) and non-homologous end joining (Park et al., 2017; B. Zhou et al., 2023) in tumor cells. At the same time LOXL2 overexpression could rescue the elevation of γH2AX levels induced by PARP9 knockdown (Supplementary Figure S5A). And immunofluorescence colocalization demonstrated that upon DNA damage, LOXL2 and γH2AX exhibited nuclear colocalization signals (Supplementary Figure S10).

### 3.5 Multidrug efflux system-related genes are highly expressed in PC and positively correlate with PARP9 and LOXL2

**FIGURE 5:**
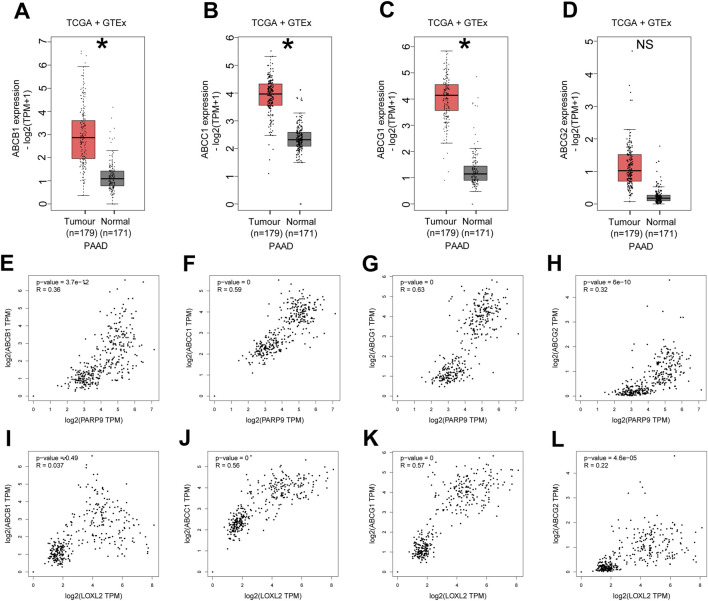
Multidrug efflux system-related genes are highly expressed in PC and positively correlate with PARP9 and LOXL2. The mRNA expression levels of ABCB1 **(A)**, ABCC1 **(B)**, ABCG1 **(C)**, or ABCG2 **(D)** in PC and normal samples were evaluated using the GEPIA online database. Correlations between PARP9 and ABCB1 **(E)**, ABCC1 **(F)**, ABCG1 **(G)**, or ABCG2 **(H)** were validated using an online database. Correlations between LOXL2 and ABCB1 **(I)**, ABCC1 **(J)**, ABCG1 **(K)**, or ABCG2 **(L)** were verified using an online database. NS, not significant. ABCB1, ATP-binding cassette subfamily B member 1; ABCC1, ATP-binding cassette subfamily C member 1; ABCG1, ATP-binding cassette subfamily G member 1; ABCG2, ATP-binding cassette subfamily G member 2. *p < 0.05.

Analysis of differentially expressed genes from our sequencing results via GO and KEGG enrichment revealed their significant involvement in ABC-type transporter activity and ABC transporters (Supplementary Figure S11). And to elucidate the relationship between PARP9, LOXL2, and drug resistance in PC, we initially performed a comparative analysis of multidrug efflux system-associated genes, including ABCB1 (ATP-binding cassette subfamily B member 1), ABCC1 (ATP-binding cassette subfamily C member 1), ABCG1 (ATP-binding cassette subfamily G member 1), and ABCG2 (ATP-binding cassette subfamily G member 2), between normal pancreatic tissues and PC tissues using the GEPIA online database. The results demonstrated that the expression levels of ABCB1 (Figure 5A), ABCC1 (Figure 5B), and ABCG1 (Figure 5C) were significantly upregulated in PC tissues compared to normal controls (all *P* < 0.05). Although ABCG2 exhibited a trend of increased expression in cancerous tissues, this difference failed to reach statistical significance (Figure 5D, *P* > 0.05). Subsequently, we further interrogated the GEPIA database to evaluate potential correlations between PARP9/LOXL2 expression and the four genes. Pearson correlation analysis revealed that PARP9 expression showed statistically significant positive correlations with ABCB1 (Figures 5E, *R* = 0.36), ABCC1 (Figures 5F, *R* = 0.59), ABCG1 (Figures 5G, *R* = 0.63), and ABCG2 (Figures 5H, *R* = 0.32) (all *P* < 0.05). In contrast, LOXL2 expression displayed no significant association with ABCB1 (Figure 5I, *P* > 0.05). However, it exhibited robust positive correlations with ABCC1 (Figures 5J, *R* = 0.56), ABCG1 (Figures 5K, *R* = 0.57), and ABCG2 (Figures 5L, *R* = 0.22), all of which were statistically significant (P < 0.05).

### 3.6 The overexpression of LOXL2 activates the multidrug efflux system and attenuates the inhibitory effects of PARP9 knockdown on it

**FIGURE 6:**
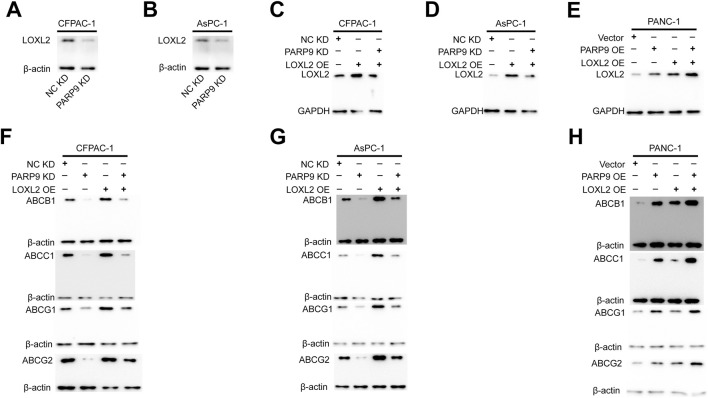
The overexpression of LOXL2 activates the multidrug efflux system and attenuates the inhibitory effects of PARP9 knockdown on it. Western blot analysis was performed to examine that PARP9 knockdown significantly decline the expression of LOXL2 in CFPAC-1 **(A)** and AsPAC-1 **(B)** cells, and the effect of LOXL2 overexpression plasmid in CFPAC-1 **(C)**, AsPAC-1 **(D)**, and PANC-1 **(E)** cells. Effects of PARP9 or LOXL2 alone or in combination on the multidrug efflux system in CFPAC-1 **(F)**, AsPAC-1 **(G)**, and PANC-1 **(H)** cells were examined by Western blot.NC, negative control; KD, knockdown; SH, short hairpin; OE, overexpressing.

To investigate the functional interplay between PARP9 and LOXL2 in PC drug resistance, we established multiple genetic manipulation models across distinct cell lines. In CFPAC-1 and AsPC-1 cell lines, LOXL2 overexpression was induced in both wild type controls and PARP9 knockdown counterparts through plasmid transfection. Conversely, parallel LOXL2 overexpression was performed in control and PARP9 overexpressing PANC-1 cells. In CFPAC-1 and AsPAC-1 cells, PARP9 knockdown significantly declined the expression of LOXL2 (Figures 6A,B), and LOXL2 overexpression plasmid significantly increased LOXL2 expression in normal control and PARP9 knockdown cells (Figures 6C,D). In PANC-1 cells, overexpressing PARP9 significantly increased LOXL2 expression, and LOXL2 overexpression plasmid significantly augmented LOXL2 expression in normal control and PARP9 overexpressing cells (Figure 6E). In CFPAC-1 and AsPAC-1 cells, PARP9 knockdown significantly decreased the expression of ABCB1, ABCC1, ABCG1, and ABCG2, and LOXL2 overexpression significantly enhanced the expression of ABCB1, ABCC1, ABCG1, and ABCG2, and LOXL2 overexpression attenuated the effect of PARP9 knockdown on the multidrug efflux system (Figures 6E,F). Simultaneous, overexpression of PARP9 and LOXL2 in PANC-1 cells significantly increased the expression of ABCB1, ABCC1, ABCG1, and ABCG2, and overexpression of LOXL2 amplified the effect of PARP9 overexpression on the multidrug efflux system (Figure 6G).

### 3.7 Gemcitabine-resistant PC cells exhibited elevated PARP9 expression and correlated with PI3K-AKT signaling pathway activation

**FIGURE 7:**
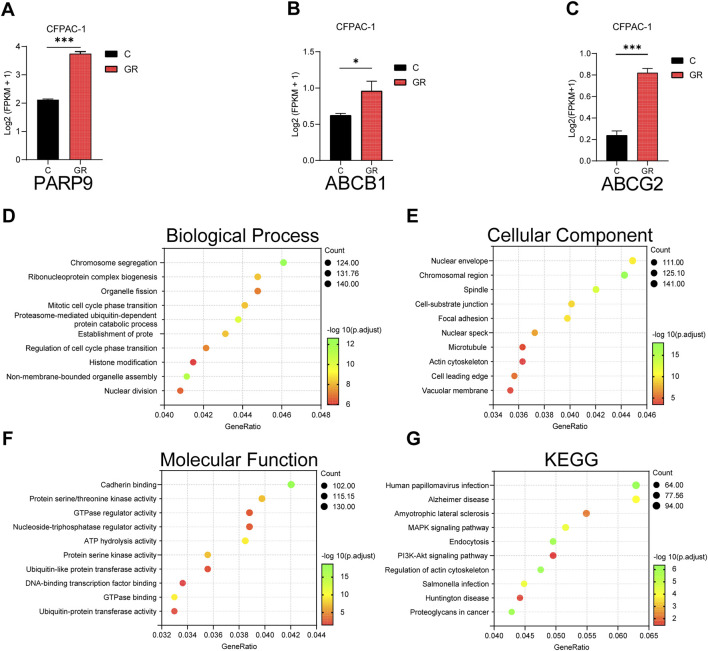
Analysis of gemcitabine resistance-induced alterations in CFPAC-1 cells using GEO dataset. The mRNA expression levels of PARP9 **(A)**, ABCB1 **(B)** and ABCG2 **(C)** in CFPAC-1 cells and GEM-resistant CFPAC-1 cells (CFPAC-1-GR) were evaluated using the GSE140077. GO analysis **(D–F)** and KEGG analysis **(G)** of differential gene. *p *<* 0.05, ***p < 0.001.

The GEO dataset (GSE140077) was analyzed to characterize gene and signaling pathway alterations in CFPAC-1 PC cells following acquired gemcitabine resistance. Results demonstrated significantly elevated expression of PARP9 in gemcitabine-resistant CFPAC-1 cells (CFPAC-1-GR) compared to parental controls (Figure 7A). Concurrently, ABCB1 and ABCG2 also exhibited marked upregulation in CFPAC-1-GR cells (Figures 7B,C). Studies demonstrate that elevated ABCB1 (An et al., 2010; Lu et al., 2019; Qian et al., 2019)and ABCG2 (An et al., 2010; Harris et al., 2014; Wei et al., 2021)expression drives gemcitabine chemoresistance in PC. Subsequent GO and KEGG enrichment analyses demonstrated differentially expressed genes were substantially participating in predominant involvement of differentially expressed genes in: chromosome segregation (Figure 7D), nuclear envelope (Figure 7E), cadherin binding (Figure 7F), and the PI3K-Akt signaling pathway (Figure 7G) which was associated with gemcitabine resistance in PC (Gu et al., 2022; Lin et al., 2023).

## 4 Discussion

Investigating PC pathogenesis and identifying effective therapeutic targets are therefore critical. While PARP inhibitors targeting PARP1 have shown promise for advanced/metastatic PC (Zhu et al., 2020), challenges including adverse effects and drug resistance limit their clinical utility (Golan et al., 2019; Kaufman et al., 2015; Lord and Ashworth, 2017; Shroff et al., 2018). PARP9, previously linked to breast cancer progression and therapy resistance (Hong, Dong, Li and Wang, 2024; Tang et al., 2018), emerges as a novel candidate warranting exploration in PC.

The PARP family proteins play crucial roles in DNA repair and apoptosis (Lehmann et al., 2015). Aberrant expression of PARP9 has been implicated in metastasis and chemotherapy resistance across various malignancies (Bachmann et al., 2014; Tang et al., 2018; Tao, Wang and Zhou, 2020). This study investigates the mechanism by which PARP9 enhances malignant behaviors in PC through LOXL2-mediated activation of the PI3K/AKT signaling pathway. We demonstrated that PARP9 is overexpressed in PC tissues. Survival and prognostic analyses revealed that elevated PARP9 expression correlates with worsened survival outcomes in patients. Knockdown of PARP9 in PC cells significantly suppressed cell growth, as evidenced by CCK-8 assays, colony formation assays, and migration/invasion experiments and concurrently inducing apoptosis, exacerbating DNA damage, and inhibiting multidrug efflux. Conversely, PARP9 overexpression markedly promoted cell proliferation and colony formation, further confirming its oncogenic role. These findings reinforce the hypothesis that PARP9 functions as an oncogene in PC, positioning it as a promising novel therapeutic target.

To further investigate the oncogenic activity of PARP9, we performed RNA sequencing, which revealed that PARP9 alteration drives significant upregulation or downregulation of numerous target genes, including the expression of DNA repair genes (BRCA1, BRCA2, XRCC2, and POLQ) being significant reduction in PARP9-knockdown cells (Supplementary Figure S12). KEGG identified the PI3K/AKT signaling pathway as the most significantly enriched pathway. Studies have demonstrated that aberrant activation of the PI3K/AKT pathway in tumors promotes cancer progression through direct modulation of nutrient transporters and metabolic enzymes, as well as regulation of transcription factors governing the expression of key metabolic components and downstream metabolic cascades (Hoxhaj and Manning, 2020). Our findings demonstrated that PARP9 activated the downstream PI3K-AKT signaling pathway by enhancing the phosphorylation of both PI3K and AKT.

In addition to cellular experiments, we established subcutaneous xenograft tumor models in nude mice using human PC cells with PARP9 knockdown or overexpression. Our results demonstrated that PARP9 knockdown significantly inhibited tumorigenesis in PC xenograft models compared with control groups, whereas PARP9 overexpression conversely promoted tumor growth.

LOXL2, a member of the lysyl oxidase (LOX) family, is overexpressed in multiple human malignancies including gastric cancer (Peng et al., 2009), hepatocellular carcinoma (Wong et al., 2014), breast cancer (Ahn et al., 2013), and squamous cell carcinoma (Peinado et al., 2008), where it correlates with aggressive clinicopathological features and poor prognosis. In PC, LOXL2 promotes gemcitabine chemoresistance (Le Calvé et al., 2016; Lee et al., 2023). Based on these studies (Alonso-Nocelo et al., 2023; Kim et al., 2024; Li et al., 2021) and our results, we believe that combination therapy targeting PARP9 and LOXL2 may be a promising strategy for the treatment of advanced and metastatic PC.

Our integrated analysis of sequencing data, bioinformatics predictions, and Western blotting revealed a potential dose-dependent correlation between PARP9 and LOXL2. Co-IP experiments confirmed their physical interaction. Functional validation showed that LOXL2 overexpression in PARP9 knockdown CFPAC-1 cells upregulated apoptosis-related proteins, DNA damage repair factors, and multidrug efflux system-related proteins compared to PARP9 knockdown controls. Notably, pharmacological inhibition of the PI3K/AKT pathway in PARP9 knockdown and LOXL2 overexpressing CFPAC-1 cells did not reduce LOXL2 expression compared to PARP9 knockdown and LOXL2 overexpressing groups, thus LOXL2 was localized upstream of PI3K/AK signaling pathway. This regulatory hierarchy aligns with established LOXL2-PI3K/AKT pathway crosstalk documented across multiple cancer types (Tan et al., 2025; S. Wu et al., 2021; J. Yang et al., 2016). And inhibition of the PI3K/AKT signaling pathway promoted apoptosis (Gao et al., 2024; Liu et al., 2021; Xu et al., 2023; Yan et al., 2020), increasd DNA damage (Iida et al., 2024; Park et al., 2017; B. Zhou et al., 2023), and suppressed the multidrug efflux system (Abruzzese et al., 2021; Bleau et al., 2009; Deng et al., 2017; El-Daly, Abo-Elfadl, Hussein and Abo-Zeid, 2023; Goler-Baron et al., 2012; Hussain et al., 2018) in PC cells. A schematic diagram illustrating the proposed mechanism was shown in Supplementary Figure S13.

The multidrug efflux system is a critical contributor to chemoresistance in cancer cells and is strongly associated with poor prognosis in malignancies (Yalcin-Ozkat, 2021). Membrane-associated ABC transporters—including ABCB1, ABCC1, ABCG1, and ABCG2—mediate drug efflux, thereby reducing intracellular drug concentrations and conferring therapeutic resistance (Dean et al., 2022; Yalcin-Ozkat, 2021; Zeng et al., 2024). Notably, PARP9 and LOXL2 enhance the expression of ABCB1, ABCC1, ABCG1, and ABCG2. Therapeutic targeting of PARP9 and LOXL2 may suppress chemoresistance in PC. Analysis of the GEO dataset revealed significantly elevated expression of ABCB1 and ABCG2 in gemcitabine-resistant PC cells compared to parental cells. ABCB1 also mediates PARPi resistance in ovarian and breast cancers (Rottenberg et al., 2008; Vaidyanathan et al., 2016). However, due to the absence of publicly available datasets on PARPi-resistant PC cells, no analysis was performed in this context.

The administration of dual-target therapeutic agents demonstrates synergistic or additive antineoplastic efficacy, thereby enabling dose reduction strategies that attenuate therapeutic dosages while minimizing treatment-associated toxicities (Bhatia et al., 2018; Y. Zhou et al., 2020). From a pharmacotherapeutic perspective, such agents present distinct advantages including optimized pharmacokinetic profiles with better pharmacokinetic profiles, greater selectivity and efficacy compared to monotherapy, and furthermore, simplified dosing regimens and reduced polypharmacy burden contribute to improved patient compliance and quality-of-life metrics (Ferro et al., 2023). To develop dual-target drugs, it is first necessary to identify protein targets with therapeutic potential and their downstream interacting proteins. In this study, we identified PARP9 as a therapeutic target of advanced PC, and identified LOXL2, a downstream interacting protein of PARP9, which is also a therapeutic target of advanced PC. Meanwhile, when PARP9 was inhibited, the lower the expression of LOXL2, the more obvious the apoptosis and DNA damage of PC cells. The findings of this study may provide ideas and evidence for the development of dual-target drugs.

We observed nuclear translocation of LOXL2 in response to DNA damage; however, direct evidence for PARP9 binding at genomic damage sites through ChIP-seq was still lacking. And we assumed that the loss of PARP9 may lead to a more closed chromatin state at the promoters or enhancers of these DNA repair genes, potentially through altering histone modifications or chromatin architecture, thereby suppressing their transcription. However, this proposed mechanism required further validation through ChIP-seq. In parallel, we reviewed key signaling components: PI3Ks represent a family of lipid kinases divided into three structurally and functionally distinct classes (types I–III), while AKT comprises three subtypes (AKT1–AKT3). Among these, PI3Kα (a type IA kinase) (Thibault et al., 2021) and AKT1 have been specifically implicated in PC (Mehra et al., 2021). It should be noted that our study did not characterize the specific subtypes of phosphorylated PI3K and AKT involved, and we encouraged further investigation into this aspect. On the molecular mechanism of ADP-ribosylation, we noted that although PARP9 alone exhibited minimal catalytic activity (Jha et al., 2009), its heterodimerization with the DNA repair factor Dtx3L markedly enhanced ADP-ribosyltransferase function, leading to site-specific mono-ADP-ribosylation of ubiquitin at Gly76 (Yang et al., 2017). While we have identified a novel PARP9–LOXL2 interaction—with LOXL2 also participating in DNA repair—it remained unclear whether this interaction stimulated PARP9’s enzymatic activity or, if so, what the relevant substrate(s) could be.

## 5 Conclusion

We elucidated the mechanism by which PARP9 influenced the proliferation of challenging cancers both *in vitro* and *in vivo*. LOXL2 was identified as a key downstream target transcriptionally regulated by PARP9, and these molecules directly interact to promote malignant PC progression. Both are overexpressed in PC cells and exhibit significant correlation with poor survival and prognosis in cancer patients. PARP9 and LOXL2 collectively drive malignant proliferation and progression in PC by modulating apoptosis, DNA damage responses, and the multidrug efflux system. Targeted suppression of PARP9 and LOXL2 expression significantly inhibited PC cell proliferation. Targeting PARP9 and LOXL2—either as monotherapy or in combination therapy—represents a promising therapeutic strategy.

## Data Availability

The datasets presented in this study can be found in online repositories. The names of the repository/repositories and accession number(s) can be found in the article/Supplementary Material.
