# Supplementary material for: PARP9 drives the malignant progression of pancreatic cancer cells by regulating apoptosis, DNA damage, and multidrug efflux systems
Source: Front Cell Dev Biol. 2025 Nov 21;13:1694345. doi: 10.3389/fcell.2025.1694345 (PMC12678316; doi:10.3389/fcell.2025.1694345)
Supplement: Supplementary file 1 [file Table1.docx]

|  |  |  |
| --- | --- | --- |
| **Supplementary Table 1.** The sequences of the primer pairs. | | |
| Gene symbol | Forward | Reverse |
| PARP9 | 5'-GGCCTCGGTGGATGGAATG-3' | 5'-GCAAACTAACCCGGATAGTCTCT-3' |
| RA3B | 5'-CCGCTATGCTGATGACACGTT-3' | 5'-ACGGTAGACTGTCTTCACCTTG-3' |
| AKAP12 | 5'-GAGATGGCTACTAAGTCAGCGG-3' | 5'-CAGTGGGTTGTGTTAGCTCTTC-3' |
| FZD10 | 5'-AGCCATCCAGTTGCACGAG-3' | 5'-GAGTCGGGCCACTTGAAGTT-3' |
| THBS2 | 5'-GACACGCTGGATCTCACCTAC-3' | 5'-GAAGCTGTCTATGAGGTCGCA-3' |
| DYSF | 5'-AAGAACAGCGTGAACCCTGTA-3' | 5'-CCTCTCGGAGTGGGACCTT-3' |
| NID2 | 5'-CCGGTGCTGTCGTCGTTAC-3' | 5'-GGCTTCGTAGAAGTGCAGGG-3' |
| CLMP | 5'-TCCTACTATGTTGGAACCTTGGG-3' | 5'-CGGTGAGCAGCCATTCAATATC-3' |
| VCAN | 5'-GTAACCCATGCGCTACATAAAGT-3' | 5'-GGCAAAGTAGGCATCGTTGAAA-3' |
| LOXL2 | 5'-GGGTGGAGGTGTACTATGATGG-3' | 5'-CTTGCCGTAGGAGGAGCTG-3' |
| MEDAG | 5'-AGGTATGTGGAACTGACCAACT-3' | 5'-TGCCTCGTGTTTACAAGAAACG-3' |
| GAPDH | 5'-GGAGCGAGATCCCTCCAAAAT-3' | 5'-GCTGTTGTCATACTTCTCATGGG-3' |

**Supplementary Table 2.** Antibody information

| Supplier, Country | Detection target |
| --- | --- |
| Proteintech, China | PARP9 (Catalog#: 17535-1-AP), XRCC1 (Catalog#: 21468-1-AP), XRCC2 (Catalog#: 20285-1-AP), and PALB2 (Catalog#: 14340-1-AP). |
| Abcam, USA | LOXL2 (Catalog#: ab314140) and ABCG1 (Catalog#: ab201776). |
| Cell Signaling Technology, USA | Caspase-3 (Catalog#: 9662), BCL-2 (Catalog#: 15071), Bax (Catalog#: 2772), phospho-PI3K (Catalog#: 4228), PI3K (Catalog#: 4292), phospho-AKT (P-AKT, Catalog#: 9271), total AKT (Catalog#: 4691), ABCB1 (Catalog#: 13978), ABCC1 (Catalog#: 72202), ABCG2 (Catalog#: 42078), and γH2AX (Catalog#: 80312). |
