# Supplementary material for: PARP9 drives the malignant progression of pancreatic cancer cells by regulating apoptosis, DNA damage, and multidrug efflux systems
Source: Front Cell Dev Biol. 2025 Nov 21;13:1694345. doi: 10.3389/fcell.2025.1694345 (PMC12678316; doi:10.3389/fcell.2025.1694345)

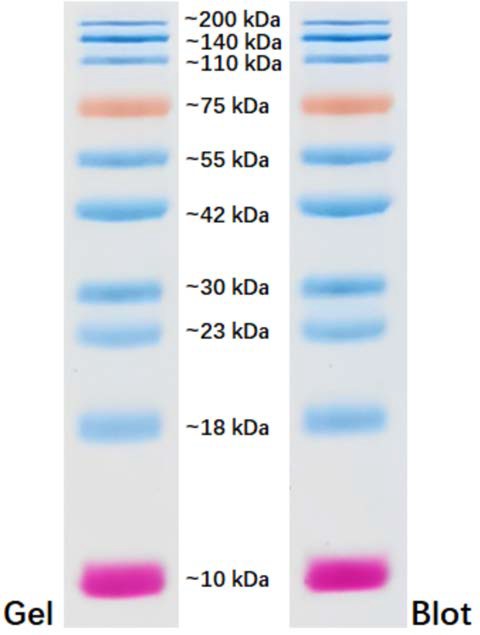


**Supplementary file 1.** All raw data from western blot.

Prestained Protein Marker II (10-200 kDa),(Servicebio, China)


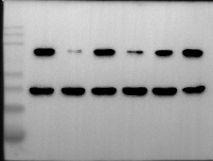

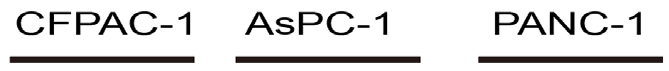

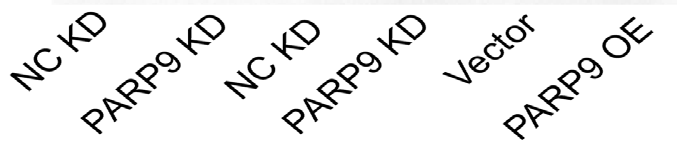

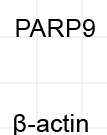

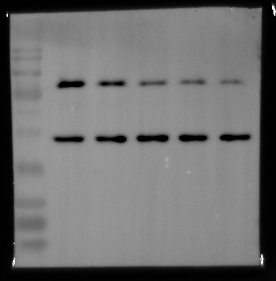

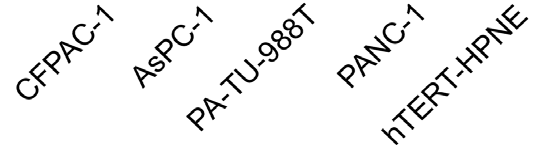

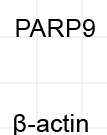

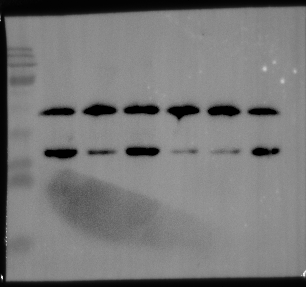

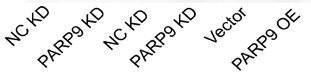

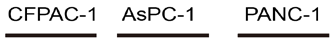


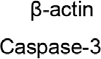

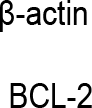


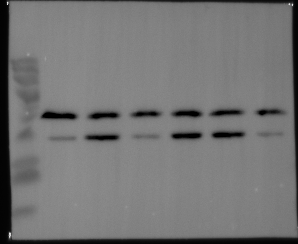

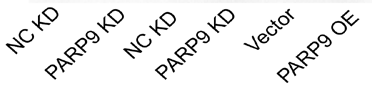

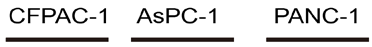

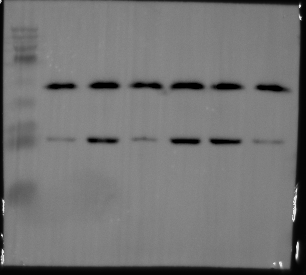

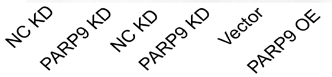

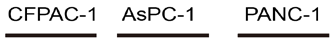

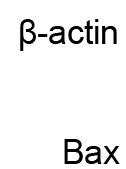

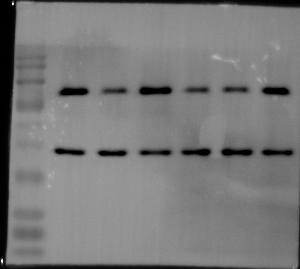

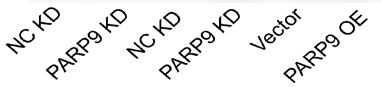

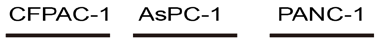

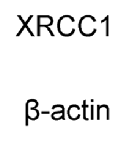


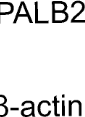


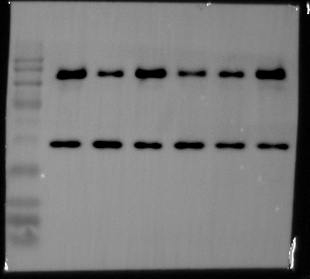

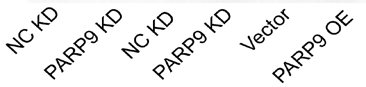

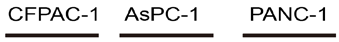

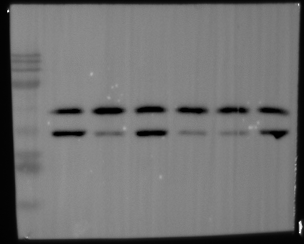

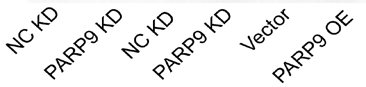

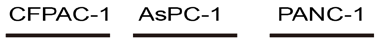

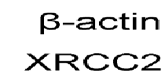


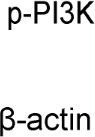

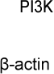


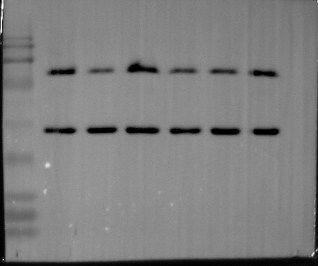

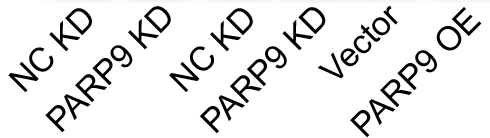

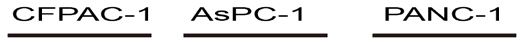

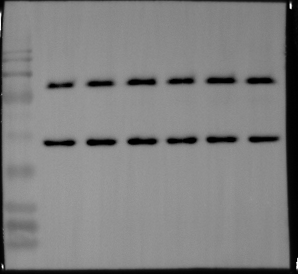

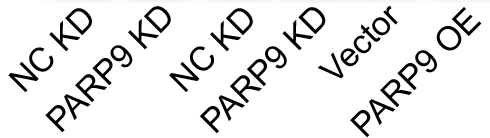

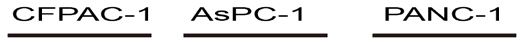

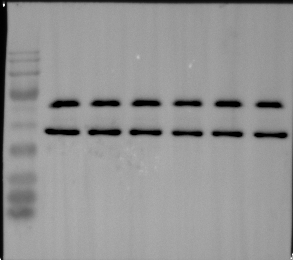

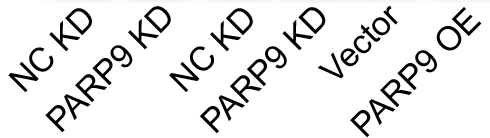

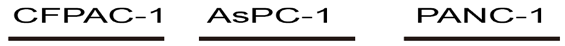


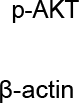

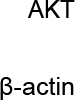


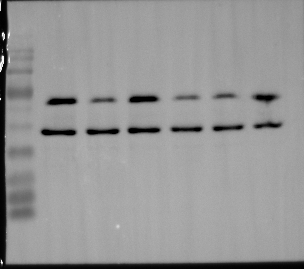

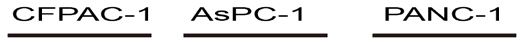

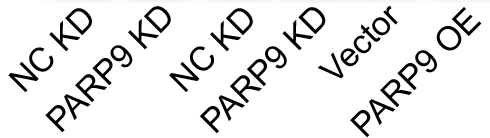

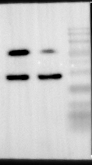

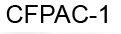

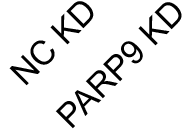

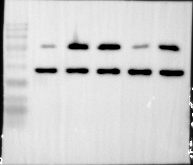

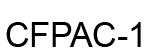

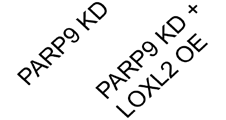

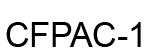

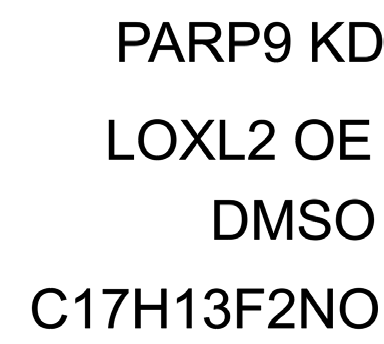

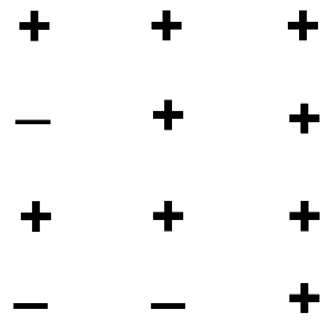

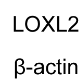

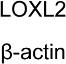


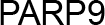


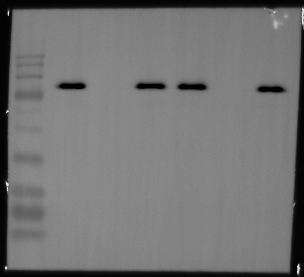

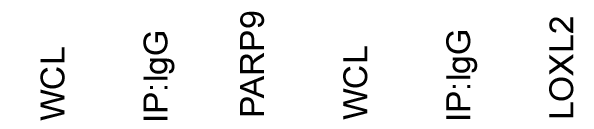

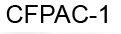

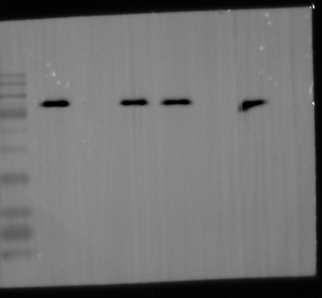

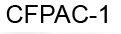

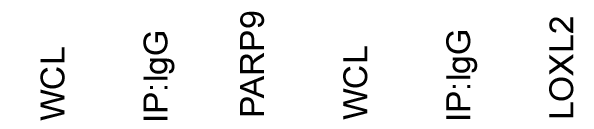

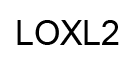

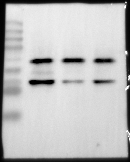

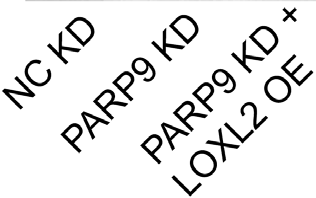

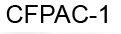


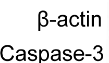

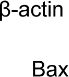

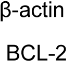


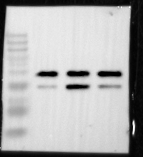

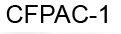

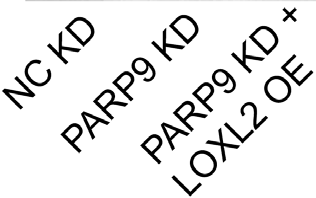

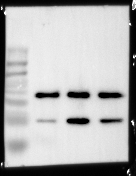

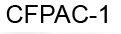

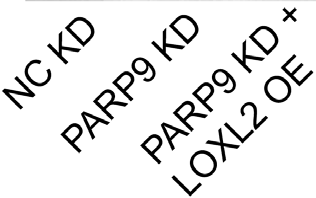

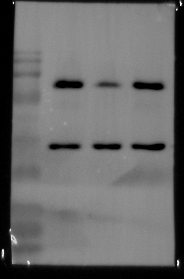

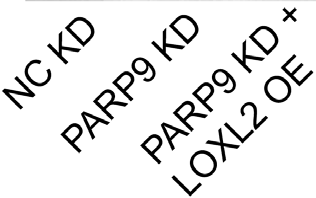

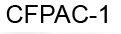


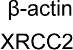

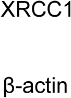


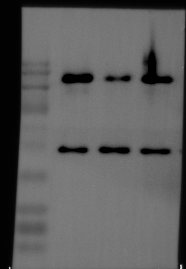

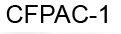

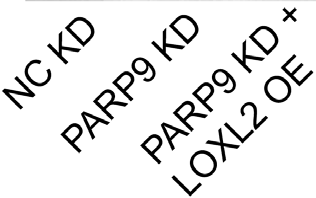

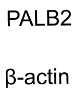

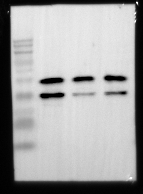

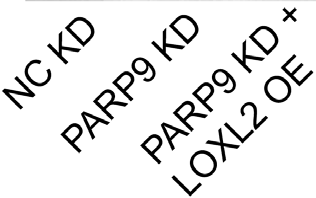

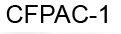


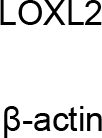

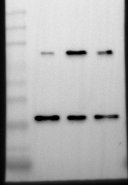

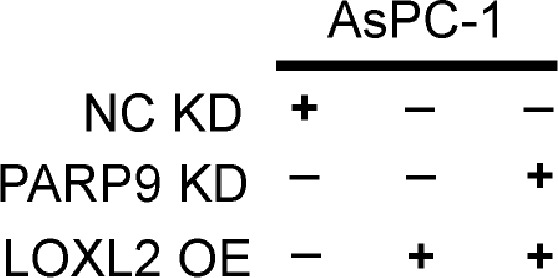

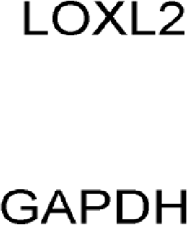


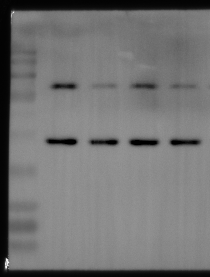

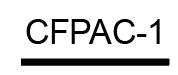

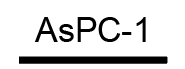

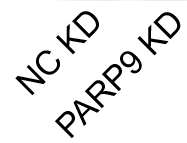

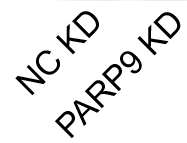

Supplement: Supplementary file 2 [file DataSheet2.docx]
