# Supplementary material for: PARP9 drives the malignant progression of pancreatic cancer cells by regulating apoptosis, DNA damage, and multidrug efflux systems
Source: Front Cell Dev Biol. 2025 Nov 21;13:1694345. doi: 10.3389/fcell.2025.1694345 (PMC12678316; doi:10.3389/fcell.2025.1694345)
Supplement: Supplementary file 3 [file DataSheet1.docx]

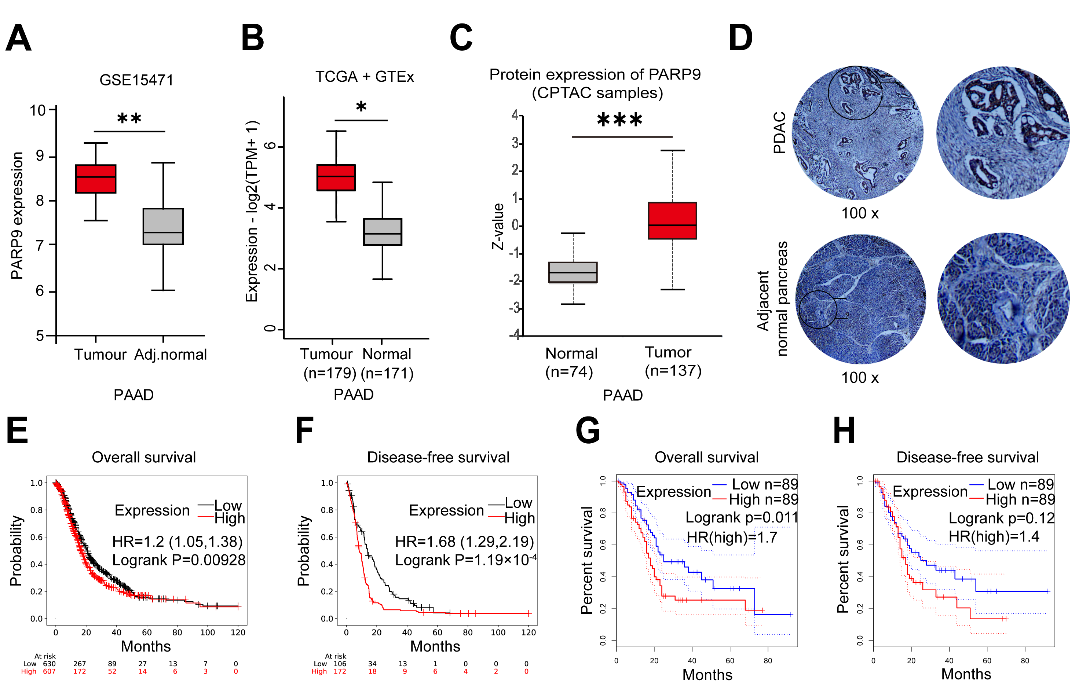
**Supplementary Fig. 1** Elevated PARP9 expression correlates with poor prognosis in PC.

The mRNA expression levels of PARP9 in PC and normal samples were evaluated using the GSE15471(A) and GEPIA online database (B). (C) The ualcan database was utilized to assess the expression of PARP9 protein in unpaired samples from CPTAC. (D) Representative immunohistochemical staining of PARP9 protein expression in PC and normal pancreatic tissue. Kaplan-Meier analysis indicated that high PARP9 expression correlated with poorer overall survival (E) and disease-free survival (F) compared to low PARP9 levels in PC patients. The relationship between the expression level of PARP9 and overall survival (G) and disease-free survival (H) in PC patients using the GEPIA online database. Experiments were repeated three times. PDAC, pancreatic adenocarcinoma. *p *<* 0.05, **p < 0.01, ***p < 0.001.

**
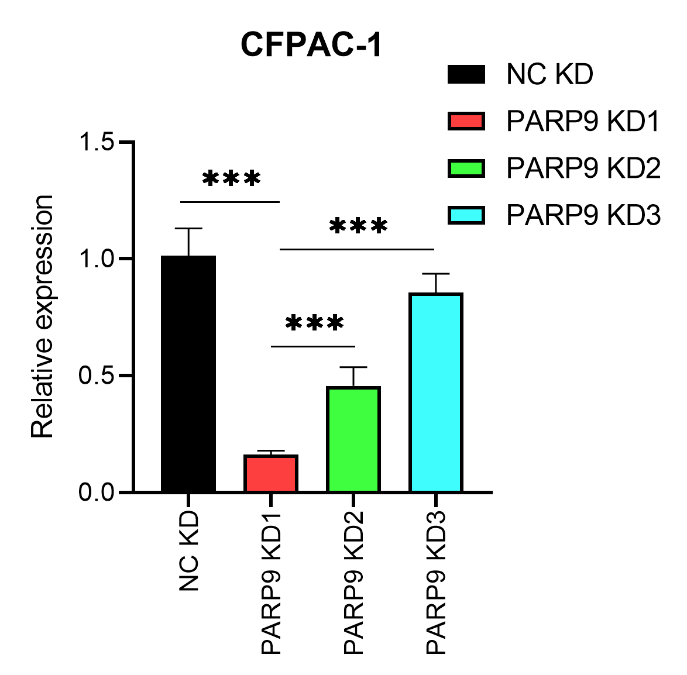
****Supplementary Fig. 2.** Validation of knockdown efficiency for three PAPA9 knockdown lentivirus (The target sequence of shRNA of PARP9 KD1 was GGGTTAGTTTGCAAGGGAAGC).

**Supplementary Fig. 3** PARP9 knockdown can suppress the malignant biological behavior in PC cells.


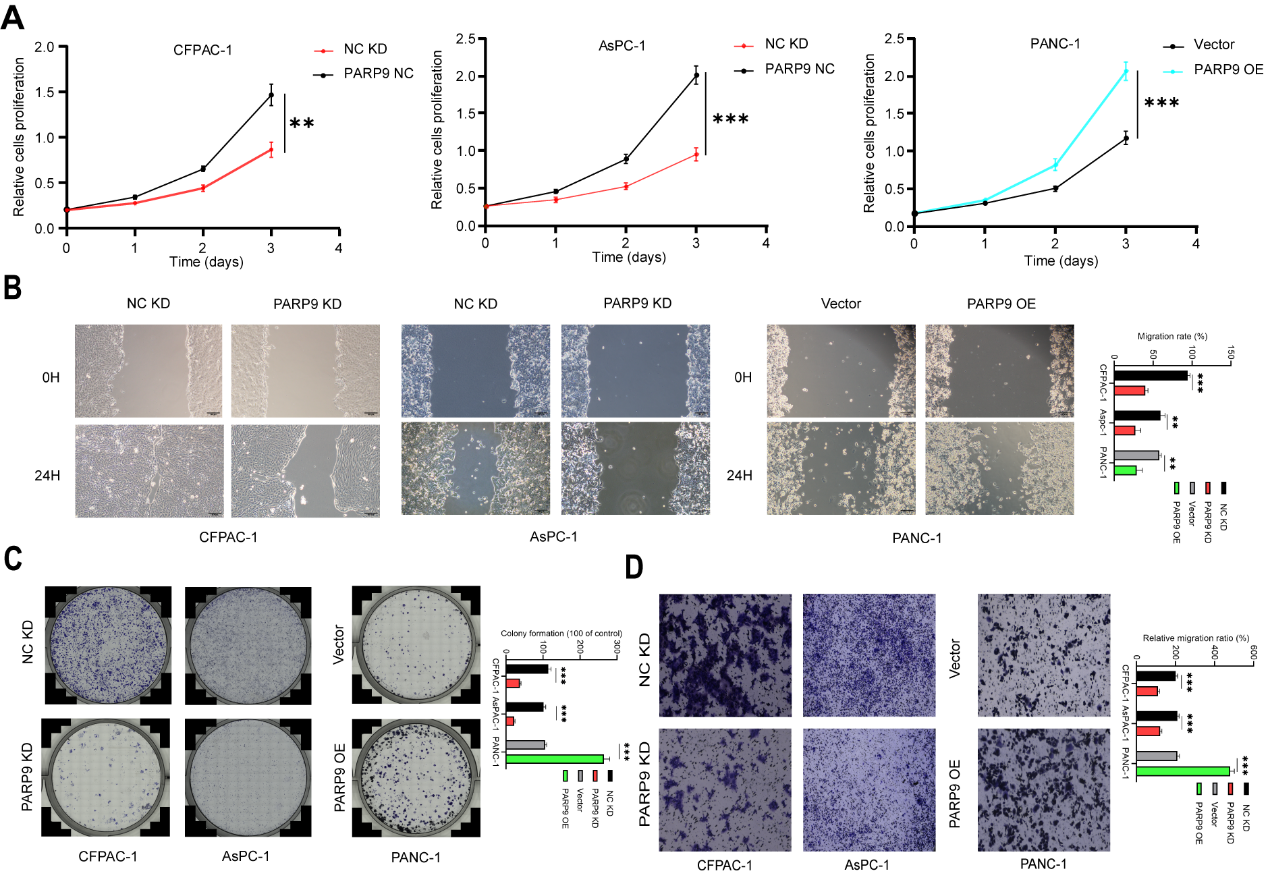


(A) CCK8 proliferation assay to investigate the effect of PARP9 changes on the proliferation ability of PC cells. (B) Wound healing experiment to investigate the healing rate of three PC cells under the influence of PARP9 after 24 hours. (C) Monoclonal formation assay to examine changes in proliferation and population-dependent ability of PC cells after altered PARP9. (D) Transwell experiment verified the change of invasion and migration ability of PC cells after PARP9 change. Bar = 200um. NC, negative control;PARP, Poly (ADP-ribose) polymerase; KD, knockdown; SH, short hairpin; OE, overexpressing. **p < 0.01, ***p < 0.001.

**
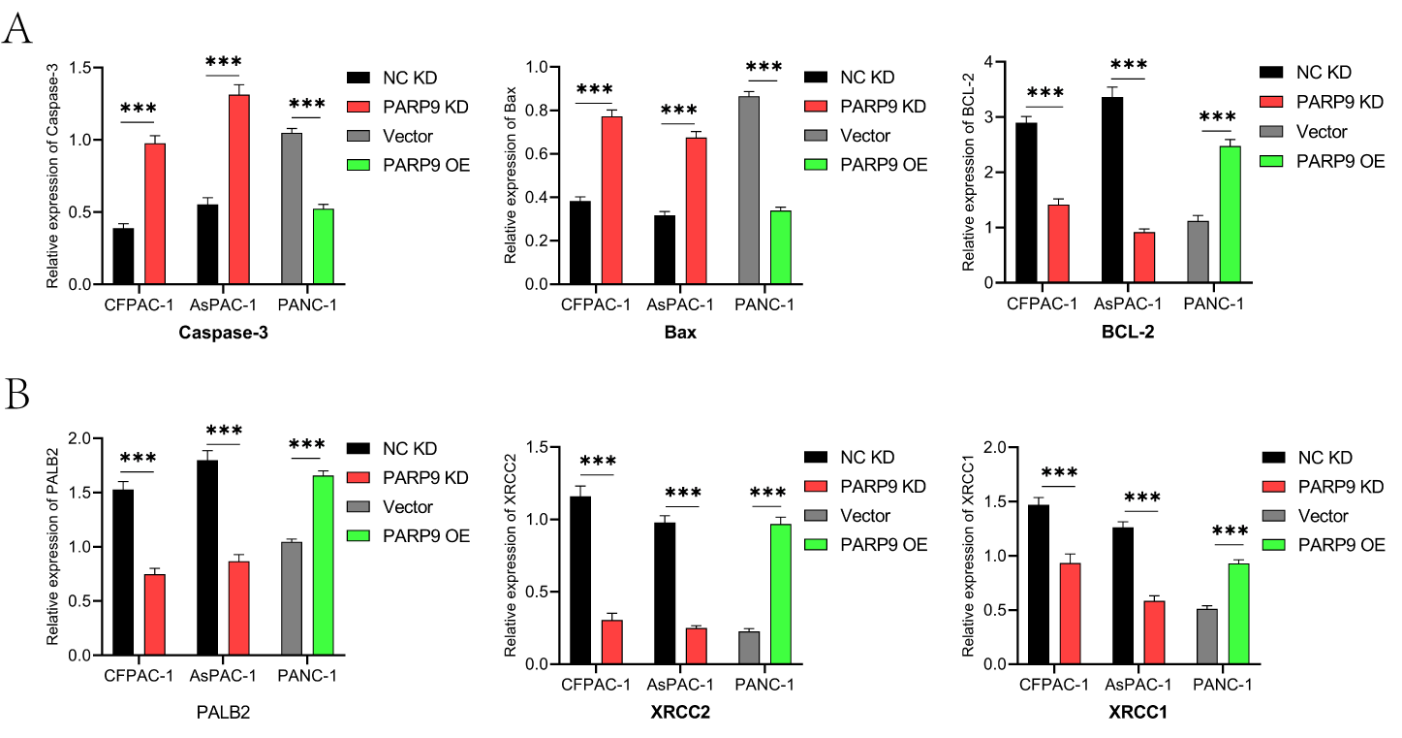
Supplementary Fig. 4.** Relative expression of Caspase-3, Bax, Bcl-2, PALB2, XRCC2, and XRCC1.

**Supplementary Fig. 5** Analysis of phosphorylated H2A histone family member X (γH2AX) by Western Blot

**
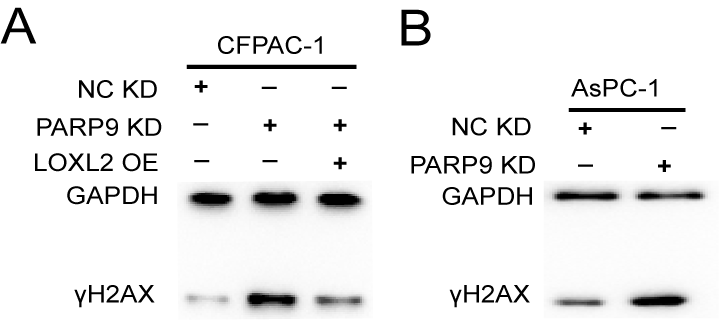
**


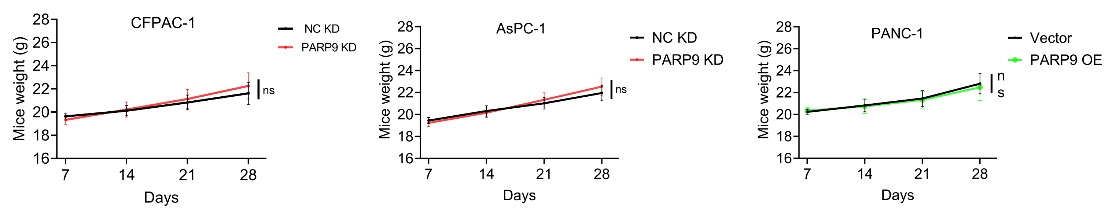
**Supplementary Fig. 6** Knockdown or overexpression of PARP9 has no effect on body weight in mice.

**Supplementary Fig. 7** PALB2, XRCC2, and XRCC1 are positively correlate with PARP9 and LOXL2 in PC.

**
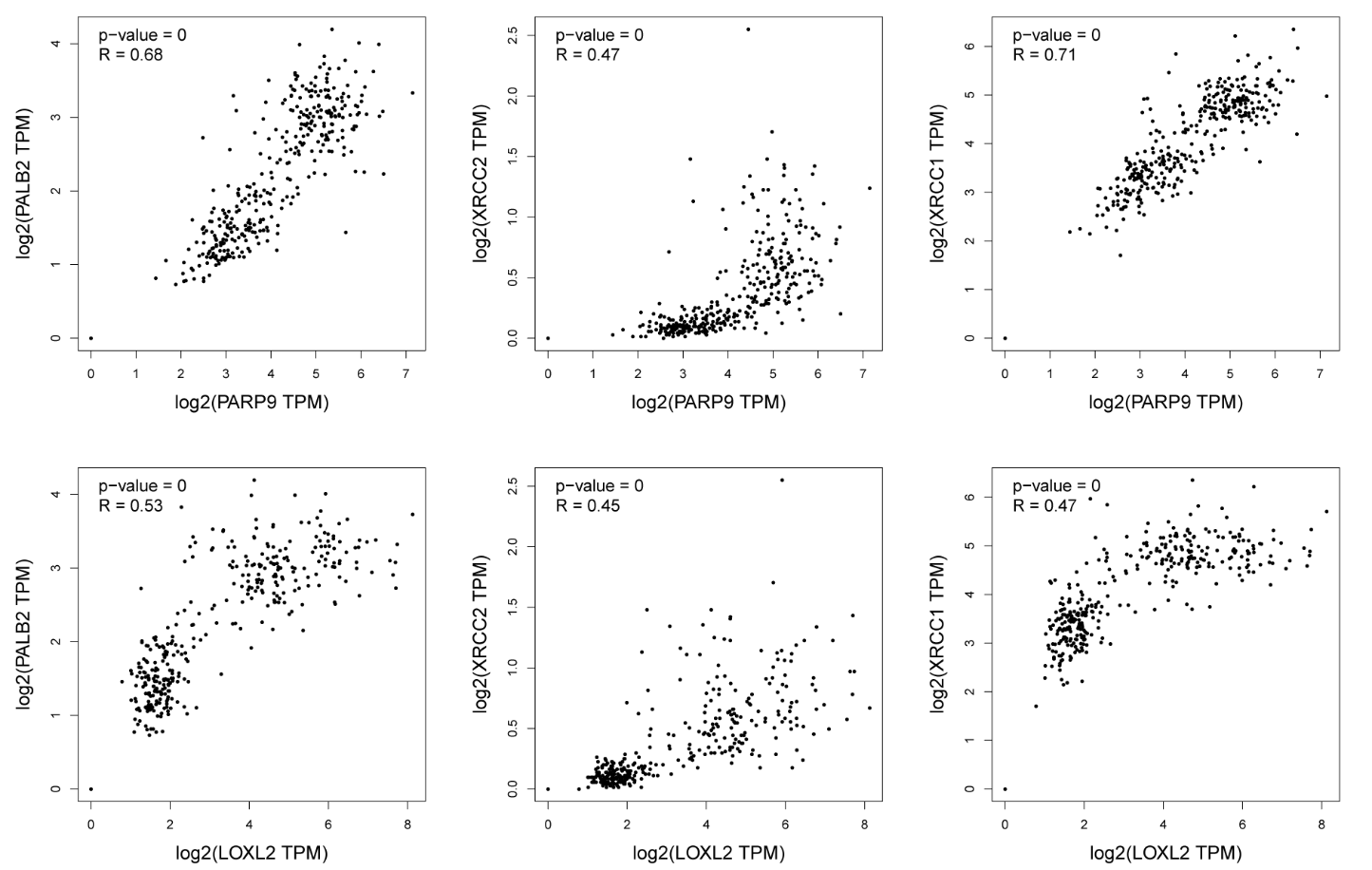
**

**Supplementary Fig. 8** BRCA1, BRCA2, RAD51, RAD54 are positively correlate with PARP9 and LOXL2 in PC.

**
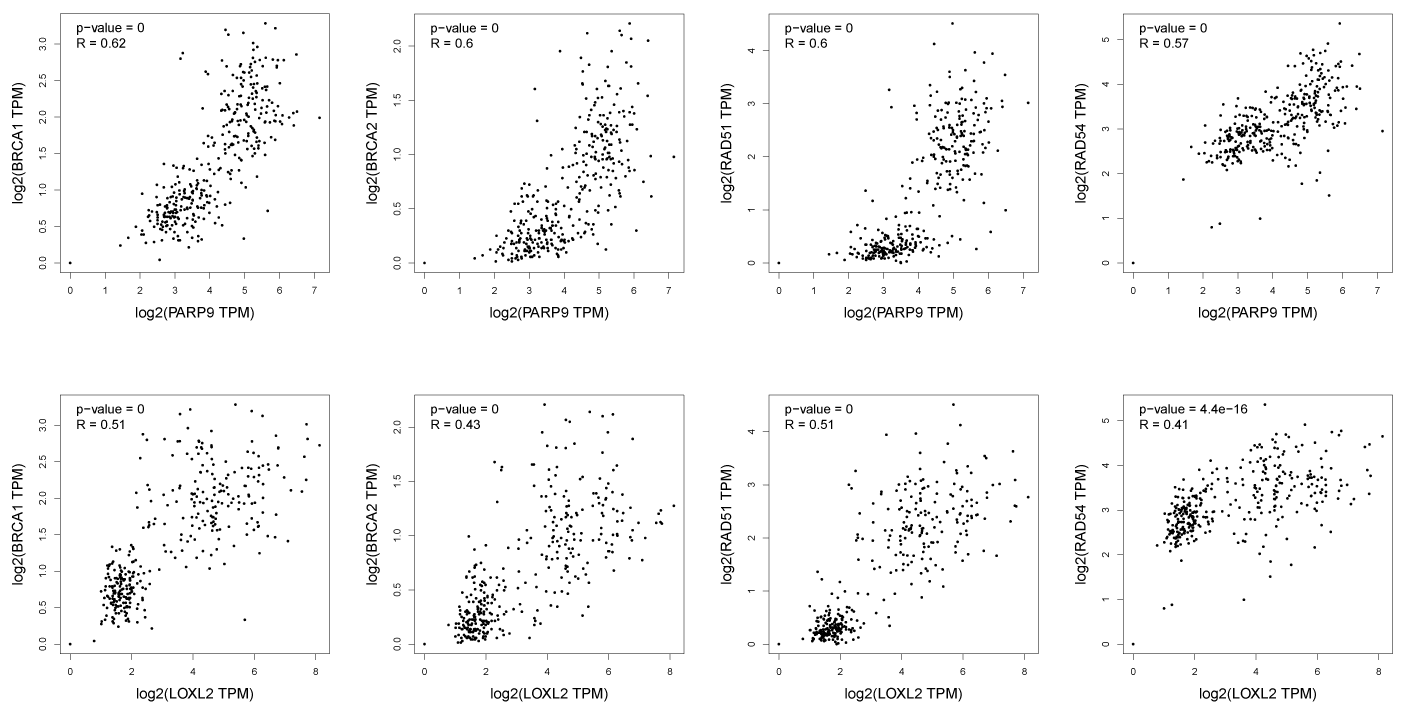
**

**Supplementary Fig. 9** KU70, KU80, DNA-PKcs, XRCC4, LIG4, and XLF are positively correlate with PARP9 and LOXL2 in PC.

**
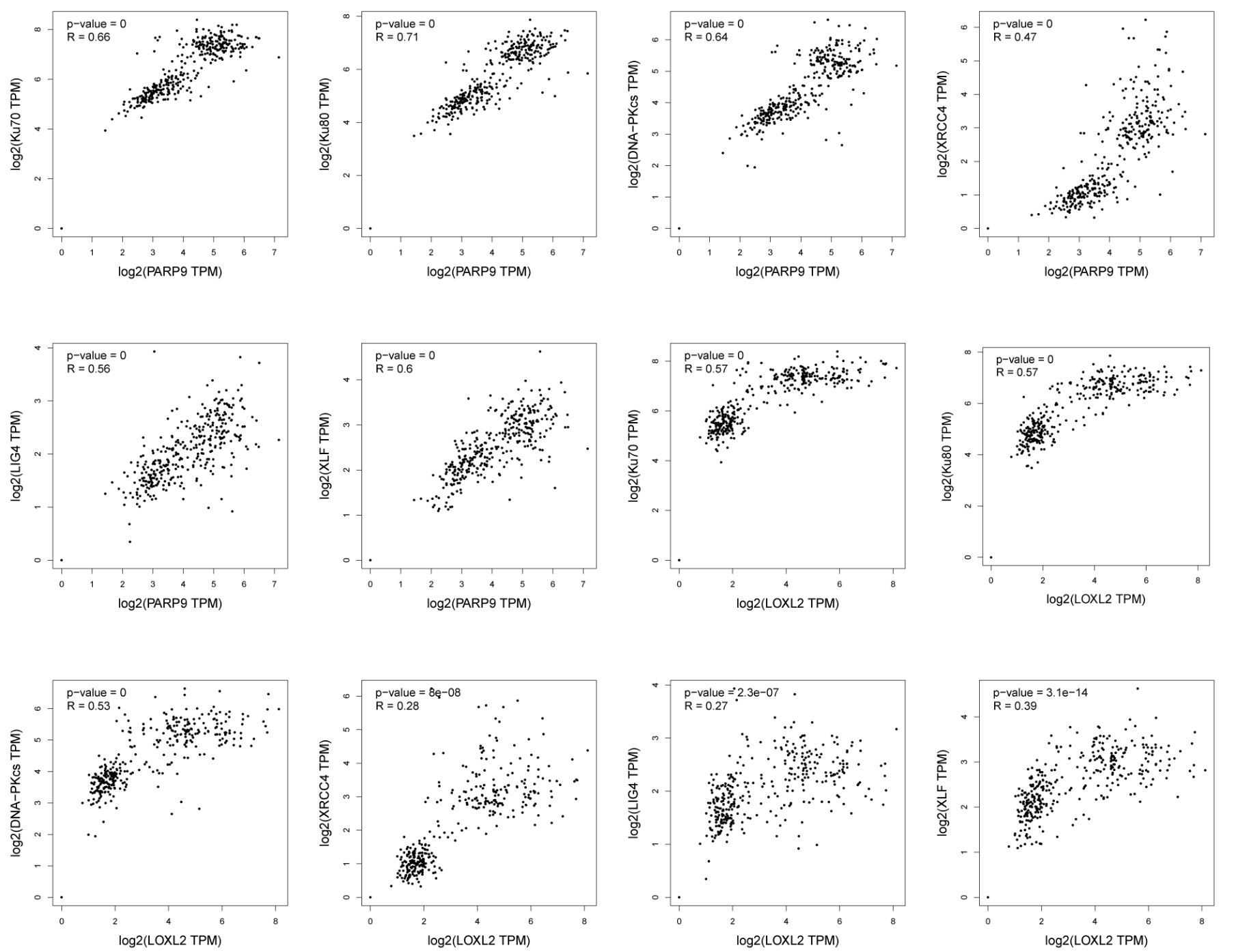
**

**
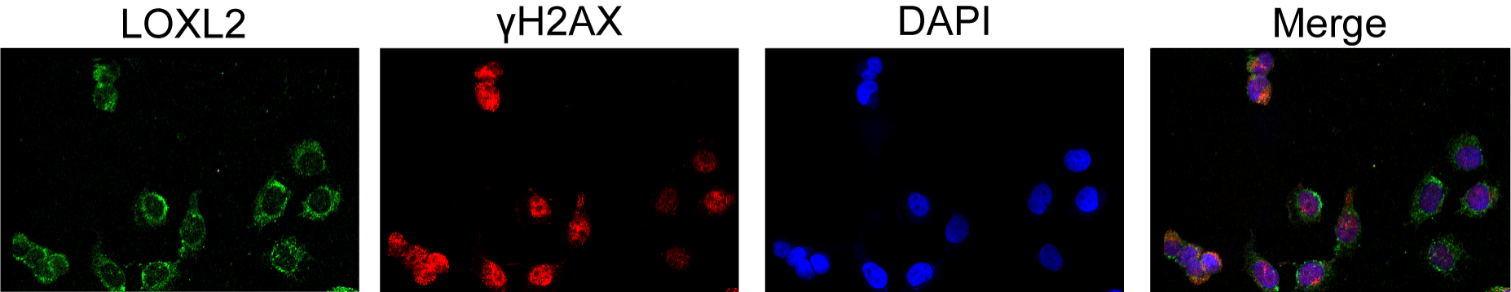
Supplementary Fig. 10** Immunofluorescence colocalization demonstrated that upon DNA damage (UV-induced), LOXL2 and γH2AX exhibited nuclear colocalization signals (CFPAC-1 PARP9 KD, 200X).


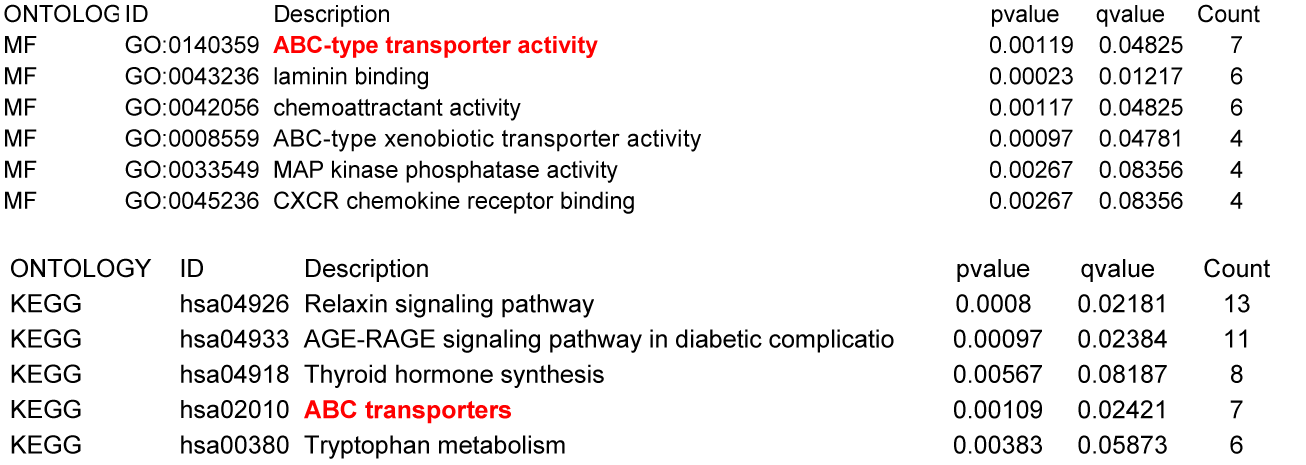
**Supplementary Fig. 11** GO analysis and KEGG analysis of differential gene.


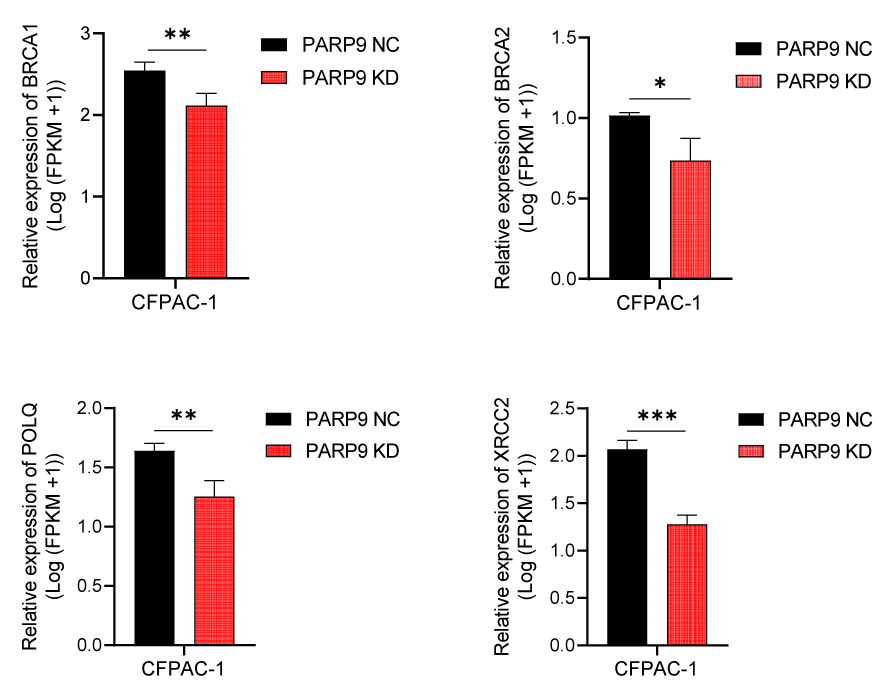
**Supplementary Fig. 12** The transcriptome sequencing results reveal that the expression levels of BRCA1, BRCA2, POLQ, and XRCC2 were significantly downregulated in the PARP9 knockdown group.

**
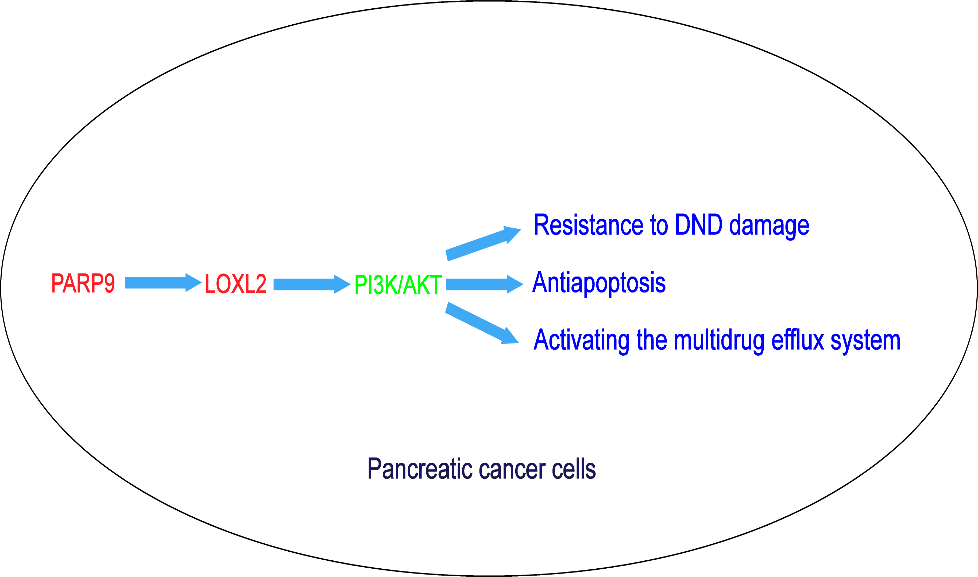
****Supplementary Fig. 13** The schematic diagram of PARP9 driving malignant progression in pancreatic cancer cells via regulation of apoptosis, DNA damage, and the multidrug efflux system.
